# Letter from Dr. Wm. Reynolds

**Published:** 1869-10

**Authors:** Wm. Reynolds


### ARTICLE VI.

### *Letter from Dr. Wm. Reynolds.*

Columbia, S. C.

Mr. Editor :—

The conditions on which the privilege of replying to the proceedings of the Odontographic Society of Pa., as published in the February number of the *Cosmos*, was conceded, were such as to induce a postponement of an answer until a more favorable opportunity and circumstances should present themselves. These are now happily found in the rapidly extending circulation of the *American Journal of Dental Science.* As a subscriber, I ask the privilege of being heard through the columns of your Journal, in defense of my improvement in the construction of Artificial Dentures on moulded base. I am the more encouraged to prefer this request from the fact that an opportunity has lately been afforded you of examining into the principles embraced in this improvement, as they were exhibited to you in skeleton models and in finished specimens.

The Odontographic Society, whose savans like Otaheitan cooks consider that "no food is fit to eat till they have chewed it,"—in other words, that no subject connected with the Dental organs or the Dental art should be permitted to pass on to the profession or the public without the stamp of their approval, has seen fit to devote a whole evening of its preuious time to an attempt to keep back from the public and from the profession, so far as its influence or the influence of the *Cosmos* might be instrumental in effecting it, one of the most important improvements of modern date in Artificial Dentistry. Ostensibly, but most disingenuously, the speakers on the occasion would have readers believe that the discussion which ensued upon the presentation of certain "beautiful specimens of art"—Reynolds' Improvement—was confined to an investigation of and pronunciamento upon their merits; yet, in no single instance, was reference made to the peculiar nature of this improvement and the intentions it was designed to fulfil. The gentlemen contented themselves with ringing the changes upon the old hackneyed subject, rubber and the Goodyear Company, oblivious to or ignorant of the fact, that the method before them was applicable, not to rubber alone, but to any moulded base now in use or hereafter to be used.

Specimens in moulded metallic base were also before them or should have been, from which it might have been observed except by those who did not choose to see, that this improvement bears a much more intimate relation to gold plate work, so highly esteemed by the Society, than to the ordinary rubber work which it so justly condemns. Had the gentlemen, who assumed the responsibility of laying without my knowledge or sanction, these specimens before this Society, taken the trouble of explaining to it, as in my absence it was his duty to do, in what way this improvement obviated every objection heretofore urged by the profession against rubber, per se, as a base, and also how effectually it removed the other no less important objections which the public entertain towards swaged plate for partial cases, the Society might have been spared the ludicrous position it now occupies. With all the gravity of "Little Peddlingtonians" the members of this Society have assumed to decide upon a matter, of which, it is more charitable to suppose them all profoundly ignorant, than that any selfish motive operated in influencing their decision.

The most prominent speakers or the occasion "saw nothing new in the specimens before them." In this they were right, since what was new and patented lay beneath the surface—concealed from view, and the vision of the members present not being more penetrating than that of ordinary people, the true nature of the improvement remained undiscovered throughout the entire debate. There are few operators, indeed, who have not at some time or other backed teeth with gold and imbedded them in rubber, but neither Dr. Nones nor Dr. Trueman have ever constructed cases on the principle carried out in these specimens. If they have, why have the profession and the public been so long deprived of its benefits? It is too late for them to look back with regret on lost opportunity. One of the objects of Patent-Laws is to bring out for the general good, inventions useful to mankind, that they be not suffered to die with the inventor, who from any motive might wish to keep them back. The masses have gone ahead of the profession in exposing the pernicious effects of swaged plates in partial cases. Few ask for them now; very few will have them. As it is with swaged metal, so it is with crude block rubber sets. The defects of these begin rapidly to force themselve upon public attention, and the deplorable practice of indiscriminate extraction is likewise becoming better understood and more generally resisted. No opposition can retard the steady progress of this improved method. The Goodyear encumbrance having ceased, as it will in a year or two, this method will be the means of recalling " our best men " to their laboratories, and of restoring the art in its perfection to their operating rooms, while the present crude style of unartistic rubber work will remain where it is, fulfilling its mission to the poor, unreproached.

Dr. Trueman, whose propensities are by nature iconoclastic, seems to care but little what source administers to his peculiar "pastime." The breaking up of old, offensive rubber sets, in which he delights to indulge, may be passed over as a matter of individual taste, but when the tendency extends itself to the breaking up of attachments of natural teeth by soldering and re-soldering the fissures caused in his gold plate by their sturdy resistance, it cannot be allowed to pass unchallenged.

As soon as a plate begins to exhibit fissure, the evidence is unmistakable that the surface from which the impression was originally taken has undergone some change, and that the time has arrived when a re-adjustment is demanded. By change of plate the existing natural organs may still be preserved; but if reinforcement after reinforcement is brought against them by repeated soldering, the merciless and unequal contest cannot long be maintained. The teeth are forced out, more slowly, it is true, but as effectually as if removed by the ready forceps of the heroic rubber worker.

In a communication entitled "An examination of a report of the American Dental Association of 1868," in the December number of the *Cosmos* for that year, the position was taken by me that the general course of argument against rubber as a base, and in favor of "gold and continuous gum only for artificial dentures;" was fallacious, and calculated to lead to error in practice.

It was there shown, that to the defects in the character of teeth specially manufactured for moulded base were to be attributed most of the evils alleged against rubber, and that swaged plate was entitled to a full share of the opprobrium which attaches to the destruction of natural organs. It was there stated that by discarding whatever has proved pernicious in both methods and combining all that is most advantageous, a denture would be constructed far more desirable than any heretofore in use. The public are entitled to the benefit arising from any improvement at the earliest moment, but unfortunately it is not always possible to reconcile the duality of sentiment which in this recently created profession is found to prevail. Theories based on sound principles, science at once acknowledges and gracefully accepts, however they may conflict with previous views; but the case is different when anything like an innovation on the *mechanique* threatens here the ordinary causes which bind the members of the several *trades* together seem to operate, and under an undefined apprehension of impending loss of prestige to their specialties, the members of dental societies not unfrequently bring themselves and their motives under the suspicion of the public. A perusal of the communication above referred to, and of the circular to be found elsewhere in this journal, is respectfully urged upon the Profession.

WM. REYNOLDS, M.D.

---

